# Prediction of Thrombus Formation within an Oxygenator via Bioimpedance Analysis

**DOI:** 10.3390/bios14100511

**Published:** 2024-10-18

**Authors:** Jan Korte, Tobias Lauwigi, Lisa Herzog, Alexander Theißen, Kai Suchorski, Lasse J. Strudthoff, Jannis Focke, Sebastian V. Jansen, Thomas Gries, Rolf Rossaint, Christian Bleilevens, Patrick Winnersbach

**Affiliations:** 1 Department of Anesthesiology, Medical Faculty, University Hospital RWTH Aachen, Pauwelsstraße 30, 52074 Aachen, Germany; jan.korte@rwth-aachen.de (J.K.); atheissen@ukaachen.de (A.T.); rrossaint@ukaachen.de (R.R.); cbleilevens@ukaachen.de (C.B.); 2 Institut für Textiltechnik (ITA), RWTH Aachen University, 52074 Aachen, Germany; tobias.lauwigi@ita.rwth-aachen.de (T.L.); lisa.herzog@alumni.fh-aachen.de (L.H.); kai.suchorski@rwth-aachen.de (K.S.); thomas.gries@ita.rwth-aachen.de (T.G.); 3 Department of Cardiovascular Engineering, Institute of Applied Medical Engineering, Medical Faculty, RWTH Aachen University, 52074 Aachen, Germany; strudthoff@ame.rwth-aachen.de (L.J.S.); focke@ame.rwth-aachen.de (J.F.); jansen@ame.rwth-aachen.de (S.V.J.)

**Keywords:** ECMO, ECLS, bioimpedance analysis, clot detection, sensor fibers, membrane oxygenator, RatOx, coagulation

## Abstract

Blood clot formation inside the membrane oxygenator (MO) remains a risk in extracorporeal membrane oxygenation (ECMO). It is associated with thromboembolic complications and normally detectable only at an advanced stage. Established clinical monitoring techniques lack predictive capabilities, emphasizing the need for refinement in MO monitoring towards an early warning system. In this study, an MO was modified by integrating four sensor fibers in the middle of the hollow fiber mat bundle, allowing for bioimpedance measurement within the MO. The modified MO was perfused with human blood in an *in vitro* test circuit until fulminant clot formation. The optical analysis of clot residues on the extracted hollow fibers showed a clot deposition area of 51.88% ± 14.25%. This was detectable via an increased bioimpedance signal with a significant increase 5 min in advance to fulminant clot formation inside the MO, which was monitored by the clinical gold standard (pressure difference across the MO (dp-MO)). This study demonstrates the feasibility of detecting clot growth early and effectively by measuring bioimpedance within an MO using integrated sensor fibers. Thus, bioimpedance may even outperform the clinical gold standard of dp-MO as a monitoring method by providing earlier clot detection.

## 1. Introduction

Since the 1970s, the use of extracorporeal membrane oxygenation (ECMO) in patients experiencing heart failure, respiratory failure, or a combination of both conditions has gained importance [1]. ECMO is considered the last therapeutic option for patients with severe acute respiratory distress syndrome (ARDS), which was particularly highlighted during the SARS-CoV-2 pandemic [2].

However, the use of ECMO is currently associated with several risks. Bleeding and thrombotic events are the most common complications associated with ECMO [3]. Blood flow through the extracorporeal circulation triggers the activation of coagulation factors and platelets via foreign surface contact, eventually culminating in consumption coagulopathy and subsequent bleeding complications. On the other side of the same coin, exposure to large foreign surfaces within the extracorporeal circulation and non-physiological shear stresses inside the pump and membrane oxygenator (MO) are the main causes of clot formation [4]. To prevent coagulation within the ECMO circuit, anticoagulation is used at the expense of increasing the risk of bleeding complications [5]. Balancing the risks of insufficient and excessive anticoagulation is difficult or even futile. There are several exacerbations originating from the effects of ECMO on coagulation, e.g., abdominal hemorrhage or disseminated intravascular coagulation where an improved monitoring of the hemostatic status would be beneficial. Yet, this study focuses on one specific subgroup: major clot formation inside the MO. Hemostatic depositions and clot growth within the oxygenator reduce gas exchange, pose the direct risk of thromboembolism to the patient, increase hemolysis, and mandate a device exchange with a major impact on outcome [6]. Typically, the median life span of ECMO systems, ranges from 7 to 10 days [7]. Continuous monitoring of anticoagulation is essential due to the risk of thromboembolism and fatal bleeding in the brain or lungs [8]. Clot formation inside the MO is the most common reason for a ECMO system exchange [9]. Existing methods for detecting coagulation events in extracorporeal circulation lack predictive early warning capabilities. An optimized early warning system for circulatory clotting holds the potential to accelerate response times and facilitate swift and thorough system exchanges. Such advancements could enhance patient safety by reducing the frequency of rushed emergency system replacements.

The prevailing monitoring method employed in clinical practice to detect blood clots in ECMO systems is the measurement of the pressure difference across the MO (dp-MO). This method is effective but lacks predictive (warning) capabilities and details on blood coagulation status.

Coagulation is a complex system with non-linear dynamics. Detrimental changes in blood coagulation status precede clot formation. Innovative real-time measurement technology may therefore be capable of predicting clot formation early enough to alert staff and prepare for an emergency system change or even preventing clot formation entirely [4]. The aim is to optimize early warning systems for detecting blood clots while also providing the localization of developing blood clots within the MO. Bioimpedance measurements inside an MO is a promising method. Bioimpedance spectroscopy (EIS) uses alternating electrical excitation to measure tissue and fluid composition [10]. For example, dielectric blood coagulometry (DBCM) is used to obtain specific information about the composition and coagulation status of blood. It assesses coagulation by monitoring alterations in erythrocyte shape, as well as the aggregation of blood components and their effect on dielectric permittivity [11]. Interfacial polarization of the erythrocyte membrane is an important effect occurring in the frequency range from a few hundred KHz to several MHz [12].

Türkmen et al. [13] recently demonstrated that bioimpedance analysis can indicate blood clot formation in a dynamic experimental test setup. It was demonstrated that clot detection using bioimpedance analysis under flow conditions in a blood-perfused test chamber is feasible. In particular, the translation of the findings to ECMO application support an earlier increase in impedance compared to dp-MO in the case of MO thrombosis.

In the present study, the test chamber was replaced with an MO equipped with sensor fibers integrated within the hollow fiber mats. Hemostasis and thrombosis detection by means of bioimpedance may differ greatly between a test chamber and an actual oxygenator. Hence, sensor fibers were integrated in a fully functional model of an MO in this study.

## 2. Materials and Methods

### 2.1. Oxygenator and Test-Circuit Design

**Figure 1:**
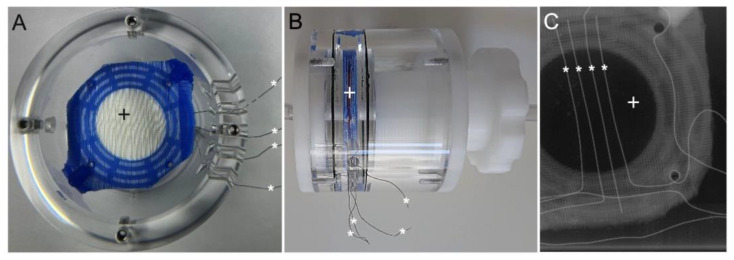
View of modified RatOx: (**A**) Top view. (**B**) Lateral view. (**C**) Axial Micro-CT scan. Hollow fiber mat bundle (+) with integrated sensor fibers (*).

To evaluate the potential of bioimpedance analysis in detecting blood clots inside a blood perfused MO, sensor fibers were integrated into a miniaturized oxygenator normally used for ECLS animal models, the RatOx [14]. The RatOx is a scaled-down model of contemporary commercial oxygenators. Its size was individually customized for the requirements of this study. Hollow fiber mat modules, comprising 11 fiber mat layers, were placed inside the oxygenator’s acrylic glass housing and fixed in place with two sealing plates. Four titanium wires (ASTM F-67 graded) were integrated between the 5th and 6th hollow fiber mat layers (Figure 1). The distance between the individual sensor fibers was 2 mm, allowing for a high-resolution measurement system. Vents in the acrylic glass cylinder at the level of the membrane fibers functioned as outlet for the impedance sensor fibers, which were then connected via crocodile clips to an impedance measuring system (AFE4300, Texas Instruments Inc., Dallas, TX, USA). Connectors inside the sealing plates enabled the connection to in- and outflow tubing. Sweep gas supply (Carbogen with 95% oxygen and 5% CO_2_) was ensured via the gas connection at the level of the sensor fibers.

**Figure 2:**
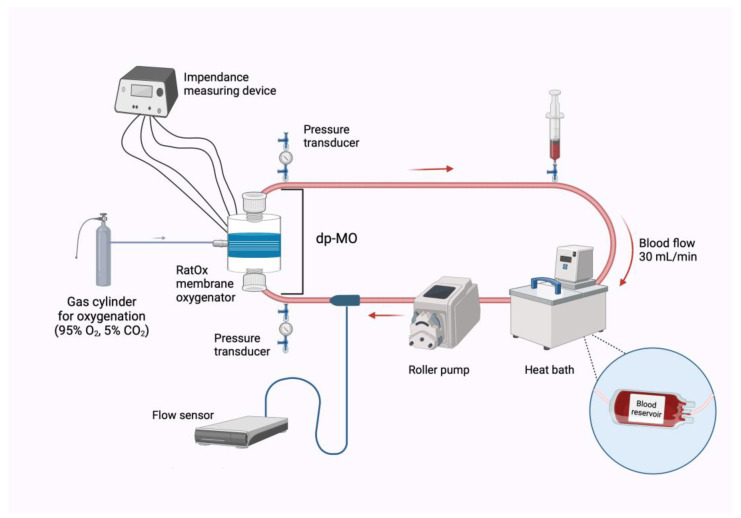
Schematic illustration of the experimental setup. dp-MO: pressure difference across the oxygenator. The syringe indicates the location of blood sampling; created with BioRender.com.

For the perfusion of the modified RatOx, an *in vitro* test circuit was designed emulating an ECMO circuit utilized in clinical settings, enabling experiments under highly realistic conditions, maximizing the fidelity of the experimental setup. The closed test-circuit (Figure 2) comprised a roller pump (CAPS double pump, Stöckert Instrumente GmbH, Freiburg im Breisgau, Germany), a blood reservoir (Standard transfusion set reservoir T3986, Fresenius Kabi AG, Bad Homburg, Germany), and the modified RatOx, all connected via PVC tubing.

In order to prevent excessive hemolysis, the roller pump was adjusted to operate with partial occlusion. To maintain a system pressure of 65 ± 5 mmHg, the flexible blood reservoir was constantly compressed using a specifically designed 3D-printed clamp device. The pressure difference was measured before and after the RatOx via 3-way stopcocks (16494C, B. Braun AG, Melsungen, Germany), measured using pressure transducers and displayed on a Datex-Monitor (Datex-Engstrom AS/3 Anesthesia Monitor, Datex-Engstrom, Helsinki, Finland). The pressure parameters were recorded at regular intervals and meanwhile closely monitored and recorded. After each blood sampling, the system pressure was restored by adjusting the clamp device, compressing the reservoir.

Constant physiological temperature was guaranteed by placing the blood reservoir inside a heat bath (Haake DC 30 Thermo Bath, Haake, Karlsruhe, Germany). In addition, a heat lamp (SANITAS SIL 16 red light lamp with 150 watts, Beurer, Ulm, Deutschland) was placed above the test circuit to avoid temperature fluctuations. The temperature was constantly measured via a standard temperature probe (TCAT-2LV Controller, Physitemp, Clifton, NJ, USA) connected with the test circuit via Luer lock. Blood flow was measured via clamp-on flow sensors (BioProTTTM Clamp-OnTM Transducer, em-tec GmbH, Finning, Germany).

### 2.2. Blood Collection from Human Donors and Filling of the Circuit

Prior to each experiment, the oxygenator was carefully primed with 7 mL NaCl (Isotonic sodium chloride solution, B. Braun, Crissier, Switzerland) to eliminate any air between the hollow fibers.

Blood was collected from healthy human volunteers after obtaining informed consent from participants and approval from the Ethics Committee of the University Hospital of the RWTH Aachen University (file no EK22-355, 24 October 2022). For each experiment, 100 mL of human blood was withdrawn via cubital venipuncture into two 50 mL syringes (8728810F, B. Braun, Melsungen, Germany) primed with heparin sodium (12626723/0319, B. Braun, Melsungen, Germany) in respective amounts. In addition, for baseline measurements, blood was directly drawn into a 3 mL citrated sample tube (05.1165, Sarstedt AG, Nümbrecht, Germany) and a 3 mL syringe (4606027V, B. Braun, Melsungen, Germany) for subsequent cell count (MEK-6500K, Nihon Kohden, Tokyo, Japan) and ACT measurement (Hemochron Jr. Signature+, Accriva Diagnostics, San Diego, CA, USA).

The test circuit was carefully filled with 93 mL of blood evacuating all air, resulting in a total filling volume of 100 mL (7 mL NaCL priming volume in the RatOx and 93 mL blood in the test circuit).

After filling the test circuit, the roller pump was started at a setting of 30 revolutions per minute (RPM), resulting in a flow rate between 28 and 32 mL/min, homogenizing blood and NaCl. The system was operated under constant temperature, flow rate, and pressure conditions. Impedance measurements were continuously recorded; dp-MO and blood parameters were recorded in constant time intervals (baseline, 5 min, 15 min, 30 min, 45 min, and 60 min).

### 2.3. Bioimpedance Measurement

The impedance measurements were carried out in the single-frequency mode of the measuring device (AFE4300EVM-PDK ,Texas Instruments Inc., Dallas, TX, USA). This analog front-end is designed for bioimpedance measurement, operating at a fixed frequency of 50 kHz, and the internal circuitry recorded at 8 Hz. The amplitude of the signal used for the 50 kHz measurement was 375 µ$A_{rms}$, ensuring that the system operated within the linear range to avoid any potential harm or polarization effects on the biological samples. The impedance measurements were calibrated with two reference resistors with measured values of 10.2 Ω and 218.5 Ω, respectively. The device’s measured voltage displayed linearity with the magnitude of impedance, allowing for the voltages from the reference resistors to establish a 2-point linear equation. This process facilitated accurate conversion of the registered voltage into bioimpedance. The four-terminal sensing method, also known as the Kelvin method, was employed for impedance measurement.

The data monitoring and acquisition were carried out using the manufacturer-provided software program (AFE4300 Device GUI, Version 1.16, Texas Instruments Inc., Dallas, TX, USA).

### 2.4. Experimental Groups, Duration, and Predefined Criteria for Termination

Two experimental groups (clotting group/control group) and their respective heparinizations were defined via preliminary experiments regarding various degrees of heparinization. The clotting group’s heparinization (0.25 I.U. heparin/mL blood) was expected to provide adequate anticoagulation to prevent immediate clotting but allow for clot formation on the oxygenator’s membrane within one hour of circulation. To ensure comparative experiments without clotting, the control group was heparinized with 5 I.U. heparin/mL blood.

The termination of each experiment was conducted using previously determined criteria indicating fulminant clot formation inside the MO. To ensure a steady flow of 30 mL/min the roller pump’s speed (RPM) was constantly adjusted. The definite termination criteria for the experiments was the doubling of the roller pump’s RPM (≥60) to sustain a constant flow, signifying impaired hemodynamics due to fulminant clot formation inside the MO.

### 2.5. Blood Sampling and Analysis

Platelet count, Activated Clotting Time (ACT), partial pressure of oxygen (pO_2_), carbon partial pressure of carbon dioxide (pCO_2_), and pH were measured at predefined time points.

Simultaneous measurements of the impedance within the oxygenator, alongside the pressure difference across the oxygenator (dp-MO) and in combination with regular monitoring of coagulation parameters, were carried out to analyze the blood’s coagulation state and to detect clot formation inside the MO.

### 2.6. Image-Based Analysis of Hollow Fiber Mats and Quantitative Evaluation Using Image J

The RatOx allows for destruction-free extraction of the fiber bundle. Post-experiment, the bundles were fixated in formalin. Macroscopic images were taken of the in- and outflow side of each hollow fiber mat bundle. Finally, the hollow fiber mats were cut out with a standardized diameter of 26 mm using a punching tool (Henkellocheisen 1400260SB, Rennsteig, Steinbach-Hallenberg, Germany) and separated from one another to facilitate macroscopic images of each layer.

The intensity of clot deposition on the oxygenator membrane was obtained via planimetric analysis of the hollow fiber mat images using ImageJ (ImageJ 1.42, National institutes of Health, Bethesda, MD, USA). The intensity of clot deposition was determined by measuring the proportion of clotted blood residues within the oxygenator. To better quantify the proportion of blood adhesion to the hollow fiber mats, color contrast analysis was carried out, dividing the scans into white and red color components.

### 2.7. Statistical Analysis

As differing durations until clot formation in each experiment led to varying endpoints, the time period 15 min before termination by fulminant clot formation was analyzed. The Anderson–Darling test was used to assess the normal distribution of impedance data. Impedance mean values of 30 s intervals within the 15 min period before fulminant clot formation were compared to baseline values, which were averaged from steady-state conditions (minute 20 to minute 15 before termination of the experiment).

The PLT count and ACT data were plotted on a relative time axis to facilitate the comparison of experiments with unequal durations. All values within the respective section were cumulated and averaged. Two-way ANOVA followed by Tukey’s multiple comparisons test was performed using GraphPad Prism software (Version 10.1.1, GraphPad Software, San Diego, CA, USA). A *p*-value of less than 0.05 was considered significant.

## 3. Results

### 3.1. Blood Clot Formation

**Figure 3:**
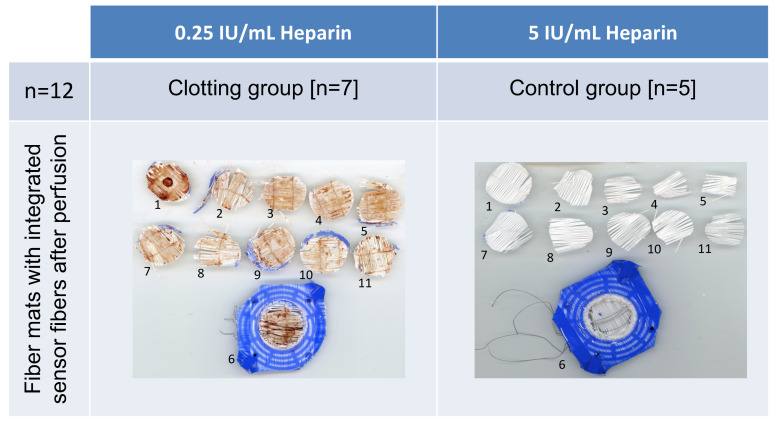
Exemplary images of stamped-out hollow fiber mats of the RatOx after flushing with sodium chloride solution. (**Left image**) Blood clot formation adherent to sensor fibers; (**right image**) no visible blood clot formation. View from inflow direction (1) until the last hollow fiber mat at the outflow region (11); one remaining hollow fiber mat (6) inside the bundle next to the sensor fibers.

Blood clot deposition between the hollow fiber mats and sensor fibers of the experimental group and control group after perfusion are shown in Figure 3.

In all seven clotting group experiments, clot deposit was optically visible to various extents on each hollow fiber mat layer. In addition, residues of blood clots adhering to the sensor fibers were evident (Figure 3, left image). In the control group, no clot formation and deposition on the hollow fiber mats occurred after 90 min of perfusion (Figure 3, right image).

**Figure 4:**
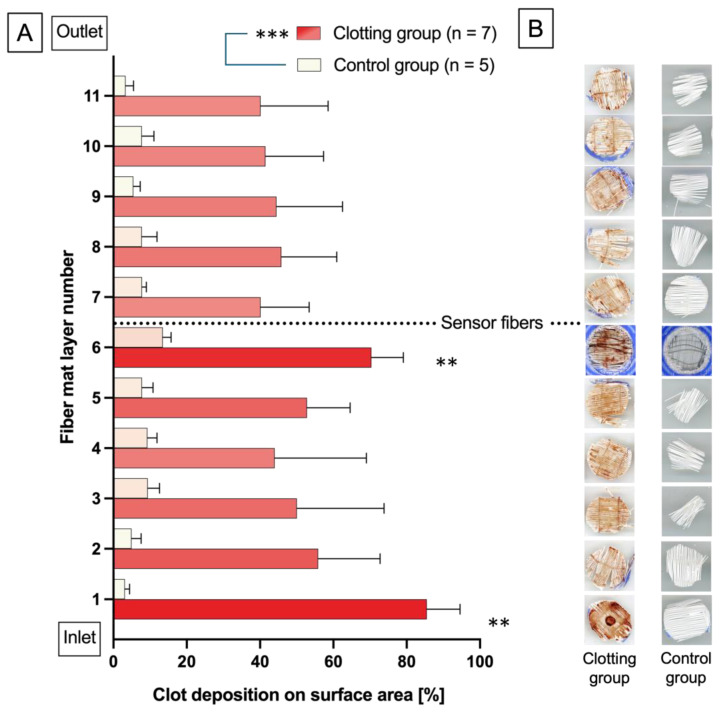
(**A**) The bar chart represents the share of hollow fiber mat area covered with blood clot residues for the clotting and control groups; the color contrast ranges from white (no/barely covered with blood clot deposits) to dark red (heavily covered with blood clot deposits) shown within the bars. Mean clot deposit on the hollow fiber mats surface is significantly higher in the clotting vs. control group and within the control group on hollow fiber mat No. 1 and 6 vs. every other hollow fiber mat in the control group. ANOVA: ** *p* < 0.002 vs. hollow fiber mat No. 2 to 5 and 7 to 11; *** *p* < 0.001 vs. control. (**B**) Exemplary scans of the hollow fiber mat layers 1–11, representative of the values shown in (**A**).

Further quantitative analysis of both the clotting and control groups regarding the amount of blood clot residue was performed through ImageJ (Figure 4). Mean total surface clot area and standard deviation (SD) for the clotting group was 51.88% ± 14.25%, and 7.28% ± 2.98% for the control group. In the clotting group, the blood clot spreads across all hollow fiber mats in all seven oxygenators, whereas the control group showed no macroscopically visible clot deposition. Analyzing the distribution of clot residues in the Clotting group, in particular the first (85.50% ± 9.08%) and sixth layer (70,38% ± 8.73%) of hollow fiber mats (numbered from blood inlet to outlet), showed the highest share of clot residues in comparison to the mean total surface clot area (51.88% ± 14.25%).

### 3.2. Pressure and Flow Compared to Impedance Measurement

**Figure 5:**
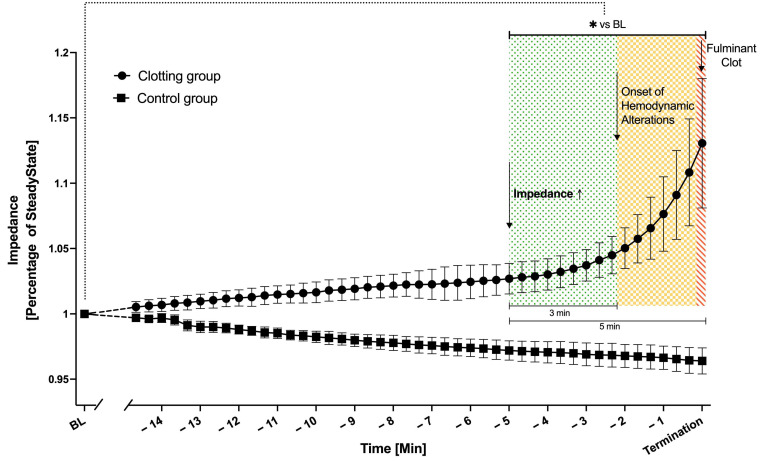
Significant increase of impedance signal 5 min prior to fulminant clot formation (green area * *p* < 0.05) in the clotting group (dots, 0.25 IU/mL heparin, *n* = 7), in contrast to the control group (squares, 5 IU/mL heparin, *n* = 5) showing no increase. First indicators for clot formation in hemodynamic parameters (increase in dp-MO) were detectable 3 min later (yellow area). Fulminant clot formation was indicated by an increase in dp-MO in conjunction with doubling of the pump’s speed (>60 RPM) resulting in the termination of the experiments (red area). Mean ± SD, ANOVA; * *p* < 0.05 vs. baseline (BL).

Due to varying durations until clot formation in each experiment, resulting in diverging endpoints, the time period 15 min before termination by fulminant clot formation was defined as the window of interest (Figure 5). The course of impedance over time was compared to baseline values, which were averaged from steady state conditions (minute 20 to minute 15 before termination). In the clotting group, impedance increased significantly 5 min prior to fulminant clot formation inside the MO and 3 min prior to first changes in dp-MO. In the control group a slight decrease in impedance was detected and no alteration in dp-MO.

### 3.3. Platelet Count and Activated Clotting Time

**Figure 6:**
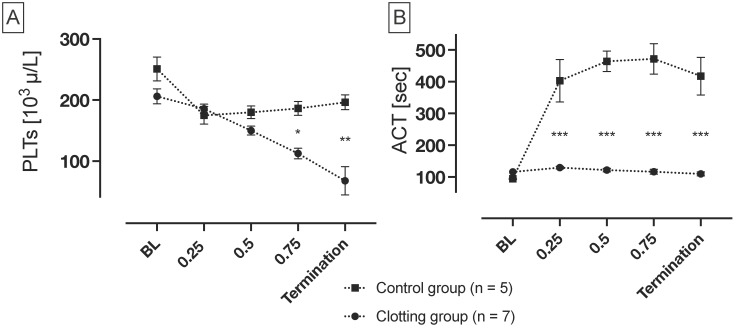
Platelet count (PLTs) and Activated Clotting Time (ACT) over the relative duration of the experiments. (**A**) PLTs in the clotting group (0.25 IU/mL; *n* = 7) decreased significantly compared to the those in the control group (5 IU/mL; *n* = 5). (**B**) In the control group, ACT increased significantly after initial heparinization, in comparison to the control group. All values within the respective quarter were cumulated and averaged. Mean ± SEM, ANOVA; * *p* < 0.05, ; ** *p* < 0.002; *** *p* < 0.001.

PLT count and ACT over the course of the experiments are displayed in Figure 6. Due to diverging endpoints of the experiments, resulting from donor–donor variabilities in coagulation properties, the time axis is presented in relative terms to facilitate better summarization and comparison of the experiments.

The PLT count in the clotting group decreased constantly from the beginning of the experiments, with the lowest PLT count observed at the end of the experiments. In contrast, the PLT count remained constant in the control group, after an initial slight decrease. As the oxygenator was built with uncoated fibers, the initial platelet drop was expected in the context of primary hemostasis. The decrease in the clotting group, compared to the control group, is significant, beginning from the last quarter of the experiment’s duration.

After heparinization, ACT slightly was elevated from baseline levels in the clotting group. In contrast, following the addition of heparin, ACT in the control group increased significantly compared to the clotting group. Subsequently, ACT maintained a constant level in both groups.

## 4. Discussion

The objective of this study was to investigate the potential of bioimpedance measurement in the early detection of blood clots within an MO perfused with human blood in a test circuit, mimicking a realistic clinical scenario. Since measurement of the pressure difference across the MO represents the gold standard in the clinics, the pressure difference over the MO was measured in comparison to bioimpedance. The experimental setup, which included a blood pump and a membrane oxygenator with integrated impedance sensor fibers, allowed for the comparison of bioimpedance analysis, pressure difference, and changes in platelets and ACT.

### 4.1. Bioimpedance Analysis as Earliest Predictor for Blood Clot Formation

The bioimpedance method, in the form of dielectrical blood coagulometry (DBCM) in connection with static human blood, has already been used in preliminary tests prior to this work [11]. In a further *in vitro* proof of concept, Türkmen et al. investigated clot formation within a blood-perfused test chamber [13]. To facilitate the transition of bioimpedance measurement for clot detection towards clinical application, the test chamber was replaced by an MO equipped with integrated impedance sensor fibers within the hollow fiber mats.

This study indicates that bioimpedance analysis within a blood-perfused MO is effective for the early detection of clot formation. A significant increase in impedance was consistent with the detection of blood clots within the MO in all cases. In contrast, the control experiments showed no blood clots after the experiments’ termination, with only slight decrease in impedance during perfusion. This decrease may be attributed to hemolysis and the subsequent increase in conductive components in the blood plasma [15].

On average, a significant increase in impedance was observed five minutes prior to fulminant MO thrombosis in comparison to the baseline measurement, whereas the dp-MO reacted significantly later. Coagulation monitoring using bioimpedance measurement inside an MO allows for the sensing of alterations in coagulation properties and the early detection of blood components’ deposition, facilitating the prediction of following clot formation and MO thrombosis. Consequently, bioimpedance measurement can offer an additional MO monitoring method, enabling the earlier detection of clots within the MO in comparison to dp-MO.

In line with these results, findings by Kaesler et al. [16] demonstrate that the dp-MO is only capable of providing reliable indications of blood clots in advanced stages. Consequently, dp-MO cannot provide a timely and accurate warning of clot formation inside the MO.

Bioimpedance measurement could be integrated as sensitive monitoring method in future ECMO systems, not only supplementing dp-MO, but possibly even replacing it. The combination of permanent simultaneous delay-free measurement of bioimpedance and dp-MO has the potential to provide higher safety levels for MOs by detecting already-growing clots instead of endpoint measurement only.

In contrast to laboratory tests, which are time-consuming and expensive, bioimpedance measurement gives real-time information about clot formation and the advantages of reliability, direct results, and no material consumption [17]. In contrast to the detection of blood clots using CT scans of the MO, bioimpedance measurement is a cost-effective and an easy-to-use method [18].

Recent advances in ECMO research aim to reduce systemic anticoagulation, thereby minimizing the risk of bleeding complications [4]. Reduced systemic anticoagulation can be enabled by enhancing local biocompatibility and hemocompatibility in critical areas, such as the MO. Improved bio- and hemocompatibility can be attained through surface modification techniques that prevent protein adsorption and platelet activation. Examples include nitric oxide-releasing coatings with additional antifouling properties [19,20] and polymer coatings [21]. Enhanced local monitoring of blood coagulation properties within the MO via bioimpedance measurement could reasonably complement the approach of improving local bio- and hemocompatibility.

It should be noted that clots are more or less equally distributed between the pump, oxygenator, and tubes [22]. In this study, the hollow fiber mat layer next to the MO’s inlet showed a significant proportion of blood clots because it was the first layer facing the bloodstream. The sixth layer also showed a significant amount of blood clots compared to other layers, as clots also formed at the sensor fibers.

Dornia et al. and Sakurai et al. [23,24] showed that clots can be localized opposite the corner of the blood inlet and outlet. Future experiments may therefore benefit from the integration of additional sensor fibers into the MO, which could provide spatially resolved information, particularly with regard to the localization of clots within the hollow fiber mats. This would permit the MO to be replaced at an earlier stage, which is currently proving to be a significant challenge in clinical practice [25]. The time saved by an early warning system for developing clots could prove to be a significant advantage in future clinical application [26]. As the impedance sensor fibers in this study were always at the level of the sixth hollow fiber mat (center of the MO), it is reasonable that not all blood clots in the entire MO were detected [27]. Follow-up studies are needed to determine where blood clots tend to form on the oxygenator membrane and where they can be detected most reliably with a high detection rate. To increase the probability of detecting blood clots, it is advisable to locate sensor fibers directly at the blood inlet of the MO or in the first hollow fiber mat proximate the inlet, as this hollow fiber mat has been proven to have a high concentration of blood clot deposition [28]. MOs used in clinical applications operate under higher flow rates and with greater blood volumes. Consequently, further studies are required to evaluate bioimpedance analysis inside MOs. To this end, additional sensor fibers for impedance measurement should be integrated within the hollow fiber mats of the MO to ensure a broader monitoring area and a more precise location of the blood clots, thus increasing the detection rate.

### 4.2. Measurement of Platelets and Activated Clotting Time

In a physiological context, blood clotting serves to protect against invading organisms after an injury, to stop bleeding and to restore homeostasis. The experimental setup used here was designed for a clotting event, which occurred during the course of the experiment due to blood contact with a foreign surface [29].

The interaction of plasma proteins with a foreign surface results in the formation of a thrombogenic surface. Proteins adsorbed on the surface subsequently interact with thrombocytes and other intrinsic coagulation factors, thereby facilitating the process of thrombosis [30].

The PLT count was intermittently monitored throughout the experiment. During the experiment, the PLT count in the clotting group declined steadily, whereas the number in the control group exhibited only slight fluctuations. The observed significant decrease in platelets can be attributed to the fact that platelets are consumed in the process of clot formation, resulting in fewer platelets measurable in non-coagulated blood. Turbulences caused by the roller pump and shear stress plausibly led to the activation of platelets and hemolysis [31]. The destruction of the cells resulted in the release of von Willebrand factor and the aggregation of GPIIb/IIIa receptors on the platelet surface, which bind to proteins adsorbed on the foreign surface [32]. Since a completely non-thrombogenic artificial surface has not yet been developed, it may be useful to monitor the possible formation of clots beyond bioimpedance measurement by continuously measuring the platelet count [33].

The ACT was employed as an additional parameter to monitor the blood’s coagulation properties within the test circuit. Alongside the activated partial thromboplastin time (aPTT), ACT is the most important parameter for monitoring heparin effect on coagulation.

The ACT in the clotting group remains constant after a slight increase after heparinization; therefore, ACT is not a reliable parameter with regard to clot development detection. In the control group, ACT was significantly higher compared to the control group due to the higher amount of heparin used.

### 4.3. Limitations

In contrast to an ECMO system used in clinical applications, this experimental setup is less complex and smaller due to its miniaturization. The hollow fiber mat bundle consists of only 11 layers, and both the priming volume and flow rate differ significantly from those used in clinical application. Furthermore, clot growth, and the subsequent obstruction and flow restriction, is less impactful and crucial in clinical ECMO systems due to the larger in- and outflow diameter, as well as its larger inner MO volume.

Even though operating the roller pump with partial occlusion ensures less hemolysis, backflow of blood could influence the extent of dp-MO, resulting in its underestimation.

The manual insertion of the sensor fibers into the hollow fiber mats introduces further inaccuracies into the measurements. In addition, the possibility of slight contamination of the fiber mats and sensor fibers cannot be entirely discounted, as they were not installed in a sterile environment.

## 5. Conclusions

In conclusion, this study demonstrates that the detection of clots by bioimpedance analysis is feasible using integrated sensor fibers for impedance measurement in a blood perfused MO. Furthermore, bioimpedance analysis revealed clot detection significantly earlier compared to pressure difference measurement, the gold standard for clot monitoring in daily intensive care unit routine, suggesting the potential for bioimpedance analysis to serve as a clot predictor rather than just a detector. Building on the previous investigation of impedance in a test chamber, it has been demonstrated that reliable early detection of coagulation within a blood circuit also exists within the hollow fiber mats of an MO.

## Data Availability

The datasets generated and/or analyzed during the current study are available from the corresponding author upon reasonable request.
